# Neonatal Transport Ventilation: Simulation to Improve Knowledge and Skills

**DOI:** 10.15766/mep_2374-8265.11272

**Published:** 2022-09-13

**Authors:** Orna Rosen, Babina Nayak, Jose Olivera, Edymel Bondal, Michelle Payne, Jillian Connors

**Affiliations:** 1 Assistant Professor, Department of Pediatrics, Albert Einstein College of Medicine; Attending Physician, Division of Neonatology, The Children's Hospital at Montefiore; 2 Assistant Professor, Department of Pediatrics, Columbia University; Attending Physician, Division of Neonatology, Harlem Hospital Medical Center; 3 Registered Respiratory Therapist, University Hospital; 4 Registered Respiratory Therapist, Division of Neonatology, The Children's Hospital at Montefiore

**Keywords:** Ventilation, Neonate, Transport, Neonatal-Perinatal Medicine, Respiratory Therapy, Simulation

## Abstract

**Introduction:**

Neonatal transport is a frequent activity in most tertiary and regional perinatal centers. Neonatal transport teams serve as mobile intensive care units and are equipped with specialized incubators that have built-in ventilators that can provide several levels of support. In our institution, we aim to educate all neonatal transport providers, including neonatal-perinatal fellows, neonatal intensive care unit–dedicated advanced practice providers, and neonatal intensive care unit–dedicated registered respiratory therapists, on transport ventilation management and troubleshooting, utilizing simulation to optimize patient care during transport.

**Methods:**

We developed scenarios based on the equipment used at our institution: an AirBorne Voyager transport incubator with a built-in Crossvent 2i+ infant ventilator, AirBorne TXP-2D high-frequency ventilator, and AeroNOx inhaled nitric oxide system (International Biomedical). Equipment and troubleshooting knowledge were assessed via knowledge tests prior to and at intervals after simulation scenario completion. We performed paired *t* tests to analyze change in test scores at each time point postsimulation compared to presimulation. Facilitated debriefing and a survey elicited feedback on learner confidence and comfort.

**Results:**

Ten learners participated in the simulations and completed the knowledge assessments. At all postsimulation time points, mean knowledge scores showed statistically significant improvements compared to presimulation scores. Feedback from learners on confidence in their skills and comfort with the equipment was positive.

**Discussion:**

Neonatal transport team ventilator knowledge and troubleshooting skills have improved after instituting this semiannual simulation training.

## Educational Objectives

By the end of this module, learners will be able to:

1. Describe the different modes of ventilation that can be administered via the transport ventilators.
2. Demonstrate how to set up the different modes of ventilation and alter settings as indicated by the scenario.
3. Identify and fix a malfunctioning ventilator (e.g., loss of pressure, empty tank, circuit leak).
4. Recognize that altering one setting in high-frequency mode (e.g., mean airway pressure) can alter other settings (e.g., frequency) and make the appropriate adjustments.
5. Assess adequate delivery of inhaled nitric oxide (iNO) clinically and based on AeroNOx monitor.
6. Describe conditions in which the amount of delivered iNO can change.
7. Identify nitrogen dioxide retention when administering inhaled nitric oxide and effectively bleed it from the ventilator circuit.

## Introduction

Interfacility transport of critically ill and preterm infants is a common practice in the United States and reflects the increase in regionalization of neonatal care. Across the country, it is not infrequent for infants to require subspecialized or higher levels of care beyond what can be provided in smaller, community-based hospitals. As experience, skill, and transport equipment continue to improve, neonatal transport teams have become mobile intensive care units and have developed the ability to provide high-level and intensive care as soon as they take over care of the patient, even before arriving back at their home institution.^1^ Infants frequently require assisted ventilation on interfacility transport due to prematurity, respiratory distress syndrome, pneumonia, and other causes of respiratory failure. Based on patient need, the team can provide a wide variety of respiratory support, from noninvasive to invasive conventional or high-frequency ventilation. Some transporters are also equipped with inhaled nitric oxide.

The makeup of neonatal transport teams varies from center to center and often reflects volume of patients requiring transport between and within institutions; some institutions staff their teams with neonatal nurses and registered respiratory therapists (RRTs) who have specialized training and experience in transport medicine, while others utilize advanced practice providers (APPs; nurse practitioners and physician assistants) or neonatal-perinatal medicine fellows.^1–4^ Regardless of the transport team's makeup and because adverse events are common during interhospital transport, it is imperative that team members have familiarity with the lifesaving equipment needed to care for these neonates.^5^ Although there is an expanding body of published work on neonatal transport team education, training remains highly variable across institutions and frequently focuses on transport physiology, medical control issues, transport workflow, communication during transport, and common procedures such as intubation and umbilical line placement.^4,6^

Simulation has been shown to be an effective teaching modality and is frequently employed to allow learners to acquire and maintain procedural competency in the neonatal intensive care unit (NICU). Simulation training has be utilized in the education of neonatal transport teams; it allows the multidisciplinary team to apply knowledge, reinforce procedural skills, manage unexpected occurrences, and improve communication in a safe, protected environment.^7–9^ Based on our literature review, there are no published simulation scenarios or educational tools that ae designed for a multidisciplinary group of learners to increase knowledge of transport ventilator equipment or that provide a forum for hands-on troubleshooting of malfunctioning equipment during neonatal transport. In our institution, we aimed to educate all neonatal transport providers, including neonatal-perinatal fellows, NICU-dedicated APPs, and NICU-dedicated RRTs, on transport ventilation management and equipment troubleshooting utilizing simulation to optimize patient care and safety.

## Methods

### Development

The neonatal transport team at the Children's Hospital at Montefiore performs approximately 200 patient transports per year. The team is composed of a NICU registered nurse, NICU RRT, and either an APP, pediatric resident, or perinatal-neonatal fellow. The team transports patients between two NICUs (level IV regional perinatal center and level III unit) and a freestanding children's hospital located on a different campus in addition to fielding transports from many referral and affiliated institutions across New York City and upstate New York.

To improve knowledge, competency, and confidence in transport ventilator management for our transport team members, from setting up the ventilator prior to transport to successfully transporting a neonate requiring intensive ventilatory support, we created several scenarios to mimic potential issues that could arise during interfacility transport. The knowledge test and scenarios were developed by a senior neonatologist (Orna Rosen) and the head of the respiratory therapy department (Jose Olivera) based on their experience and expertise. We conducted in situ simulation sessions with NICU APPs, perinatal-neonatal fellows, and NICU RRTs. We utilized our transport incubator and ventilators attached to an artificial lung to simulate functioning and malfunctioning equipment. There were no prerequisites or prework for the participants. We assessed knowledge via a knowledge test that was given immediately prior to the simulation session and then at 1, 4, and 8 weeks after the session. We repeated the simulations 3 months after the first session and administered the knowledge test 1 and 4 weeks later. We elicited confidence with a brief survey and via facilitated debriefing at the completion of the simulation sessions.

### Equipment

**Figure 1.**
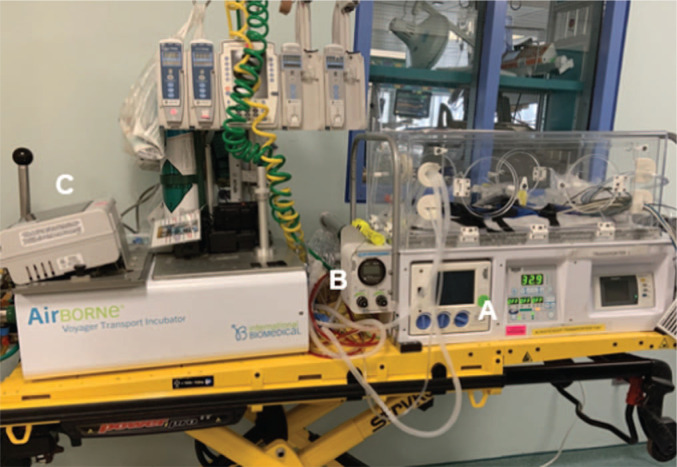
Transport equipment used at our institution: AirBorne Voyager transport incubator with (A) built-in Crossvent 2i+ infant ventilator, (B) AirBorne TXP-2D high-frequency ventilator, and (C) AeroNOx inhaled nitric oxide system (International Biomedical). Photo is owned by the authors.

Equipment required by the simulations included the following:

• AirBorne Voyager transport incubator with a built-in Crossvent 2i+ infant ventilator, AirBorne TXP-2D high-frequency ventilator, and AeroNOx inhaled nitric oxide system (International Biomedical; Figure 1).
• Conventional ventilator circuit.
• High-frequency ventilator circuit with Phasitron.
• Artificial lung.

### Personnel

The learners were neonatal-perinatal fellows, NICU-dedicated APPs (nurse practitioners and physician assistants), and NICU-dedicated RRTs. The simulation was facilitated by an attending neonatologist, who described the scenarios, and a senior RRT, who adjusted the ventilators, tanks, and circuit connections based on the different scenarios.

### Implementation

The simulation sessions took place in a room in the NICU separate from patient care areas to allow the learners to focus and to limit distractions. A simulation center was not used as the specialized transport equipment was not available there. The sessions occurred during the neonatal-perinatal fellows’ dedicated education time. The facilitators remained in the room during the scenarios. Five vignettes were completed during each session (Appendix A). Each vignette described the initial transport ventilator setup and the proposed malfunction that the learners were expected to identify, troubleshoot, and correct. The scenarios outlined the potential reasons for each malfunction and listed the applicable corrective actions. A visual guide for each of the vignette's ventilator settings was provided to assist the facilitator in recreating the settings (Appendix B). Each scenario took approximately 3–5 minutes to complete and was followed by a 5- to 10-minute debrief. In total, the session took approximately 1 hour.

### Debriefing

The attending neonatologist and senior RRT conducted debriefs after each scenario. They asked the learners to summarize the scenario, describe the nature of the ventilator malfunction, and explain how they went about troubleshooting and addressing the issue. In addition, confidence and comfort with ventilator management during transport and troubleshooting were elicited by open-ended questions from the facilitator and a brief, five-question survey completed on paper by the learners at the end of the session (Appendix C).

### Assessment

Knowledge of transport ventilation and troubleshooting was assessed utilizing a 19-item knowledge test (multiple-choice and open-ended questions; Appendix D). The knowledge test was completed by participants prior to completing the session and 1, 4, and 8 weeks after the simulation session to assess knowledge retention. Troubleshooting skills were also assessed by direct observation during the simulations. At the end of the simulation session, the facilitator reviewed the knowledge test answers (listed in Appendix E) with the learners as a group. The learners were not given their test scores.

### Statistical Analysis

Mean scores with standard deviations were calculated for the pretest and at each time point postsimulation. We performed paired *t* tests to analyze change in knowledge test scores at each time point postsimulation compared to presimulation. We used the Bonferroni procedure to adjust for multiple testing. Two-sided *p* values less than .05 were considered statistically significant.

## Results

**Figure 2.**
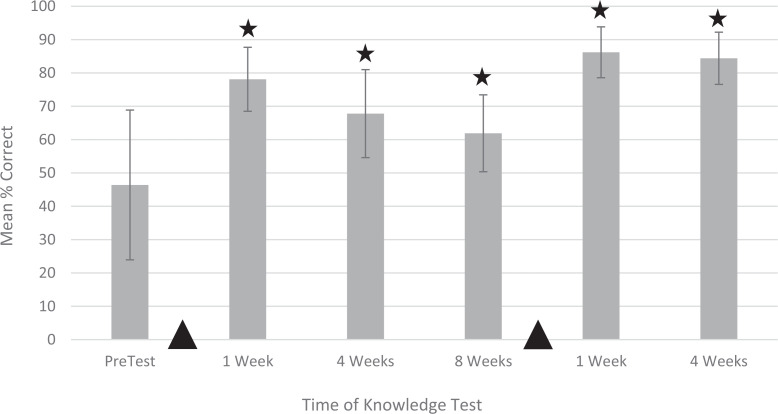
Learner performance on the 19-item knowledge test given prior to the first simulation session and at 1, 4, and 8 weeks afterward. The session was repeated, and the knowledge test was readministered 1 and 4 weeks afterward. Dark triangles denote timing of the simulation sessions. Each bar represents mean percentage correct out of a maximum score of 100%. Error bars indicate standard deviations. Stars denote *p* values less than .05.

Ten learners participated in the simulations and completed the knowledge assessments. Neonatal-perinatal fellows of all training years, NICU-dedicated APPs, and NICU-dedicated RRTs were represented in the group. At all postsimulation time points, mean knowledge test scores showed statistically significant improvements compared to presimulation scores (unadjusted *p* < .001 at 1 week postsimulation, *p* < .001 at 4 weeks postsimulation, *p* = .008 at 8 weeks postsimulation, *p* < .001 at 1 week after the second simulation, and *p* < .001 at 4 weeks after the second simulation; Figure 2). After applying the Bonferroni procedure, results remained statistically significant.

Feedback elicited during facilitated debrief and via a brief survey at the conclusion of the simulation scenarios was overwhelmingly positive. According to the postsession survey, the learners strongly agreed or agreed (scores of 1 or 2, respectively, on a 5-point Likert scale with 1 = *strongly agree* and 5 = *strongly disagree*) that the sessions were highly relevant to their roles as NICU providers and that they were confident in their troubleshooting skills after completing the scenarios.

## Discussion

We utilized simulation in a novel way not previously described in the literature to improve team member knowledge of transport ventilator equipment, ventilator management, and hands-on troubleshooting skills. These scenarios were unique because a traditional simulation manikin was not used. Instead, the transport ventilator systems were set up as they would have been with an actual patient and then were attached to an artificial lung to mimic normal functioning and provide true-to-life feedback when malfunctioning. In this way, the transport team became more familiar with standard ventilator setup and the many different alerts and alarms that could occur while ventilating an actual patient.

Knowledge as reflected by scores on the knowledge test we developed for these scenarios increased significantly after performing the simulation. We noted a trend towards lower scores as time from simulation session increased. This decay in knowledge retention over time highlighted the importance of periodically repeating the simulation session. After repeating the simulation session 3 months following the first implementation, knowledge scores were maintained at a higher level. While RRTs would be expected to perform well in these scenarios as they were most familiar with the transport ventilator equipment, we felt that it was critical that all team members have the knowledge and skill to manage and troubleshoot the equipment in order to maximize the safety of neonatal transport and limit adverse events.

As a result of the feedback elicited from the learners, transport simulation has been incorporated into the neonatal-perinatal fellow training program's simulation curriculum where it is conducted every 4–6 months. It has also been added to the first-year fellow orientation. In this way, fellows have the opportunity to improve their transport ventilation knowledge and troubleshooting skills several times during their training.

### Limitations

There were several limitations to our project. Our NICU is medium-sized and staffed with eight full-time APPs and several NICU-dedicated RRTs, and our fellowship training program is small to moderate in size (six fellows total). As a result, our sample size of learners completing the pretest, both simulations, and all posttests was small. Because the sample size was small, NICU role (i.e., fellow, APP, RRT) was separated from knowledge assessment score to maintain anonymity. Also, we have moderate transport volume. Because of this, the leaners had varying levels of transport experience prior to the first simulation session, ranging from little to none (fellows early in their training) to established proficiency (neonatal nurse practitioners with more than 10 years’ experience). Despite this, scores on the knowledge test still significantly improved postsimulation. This highlights that NICU transport practitioners of all levels and disciplines can benefit from this simulation training. In addition, we did not give the learners their knowledge test scores. We wanted the learners to focus more on hands-on troubleshooting skill acquisition rather than written test performance.

There are many different types of transport ventilators and equipment. The simulation scenarios described in this resource are based on the specific equipment we use at our institution, including high-frequency transport ventilation and the ability to provide inhaled nitric oxide during transport. Broader applicability is limited by the equipment utilized at other NICUs. However, the scenarios and assessment tools we developed can be modified based on the equipment utilized and can be tailored for use in other institutions.

## Appendices


Simulation Scenarios Guide.docxTransport Ventilator Troubleshooting Visual Aid.pptxPostsession Survey.docxLearner Knowledge Test.docxKnowledge Test Answers.docx
*All appendices are peer reviewed as integral parts of the Original Publication.*
