# Supplementary material for: Neonatal Transport Ventilation: Simulation to Improve Knowledge and Skills
Source: MedEdPORTAL. 2022 Sep 13;18:11272. doi: 10.15766/mep_2374-8265.11272 (PMC9468152; doi:10.15766/mep_2374-8265.11272)
Supplement: Supplementary file 1 — Simulation Scenarios Guide.docxTransport Ventilator Troubleshooting Visual Aid.pptxPostsession Survey.docxLearner Knowledge Test.docxKnowledge Test Answers.docx [file mep_2374-8265.11272-s001.zip › B. Transporter Ventilator Troubleshooting Visual Aid.pptx]

## Slide 1
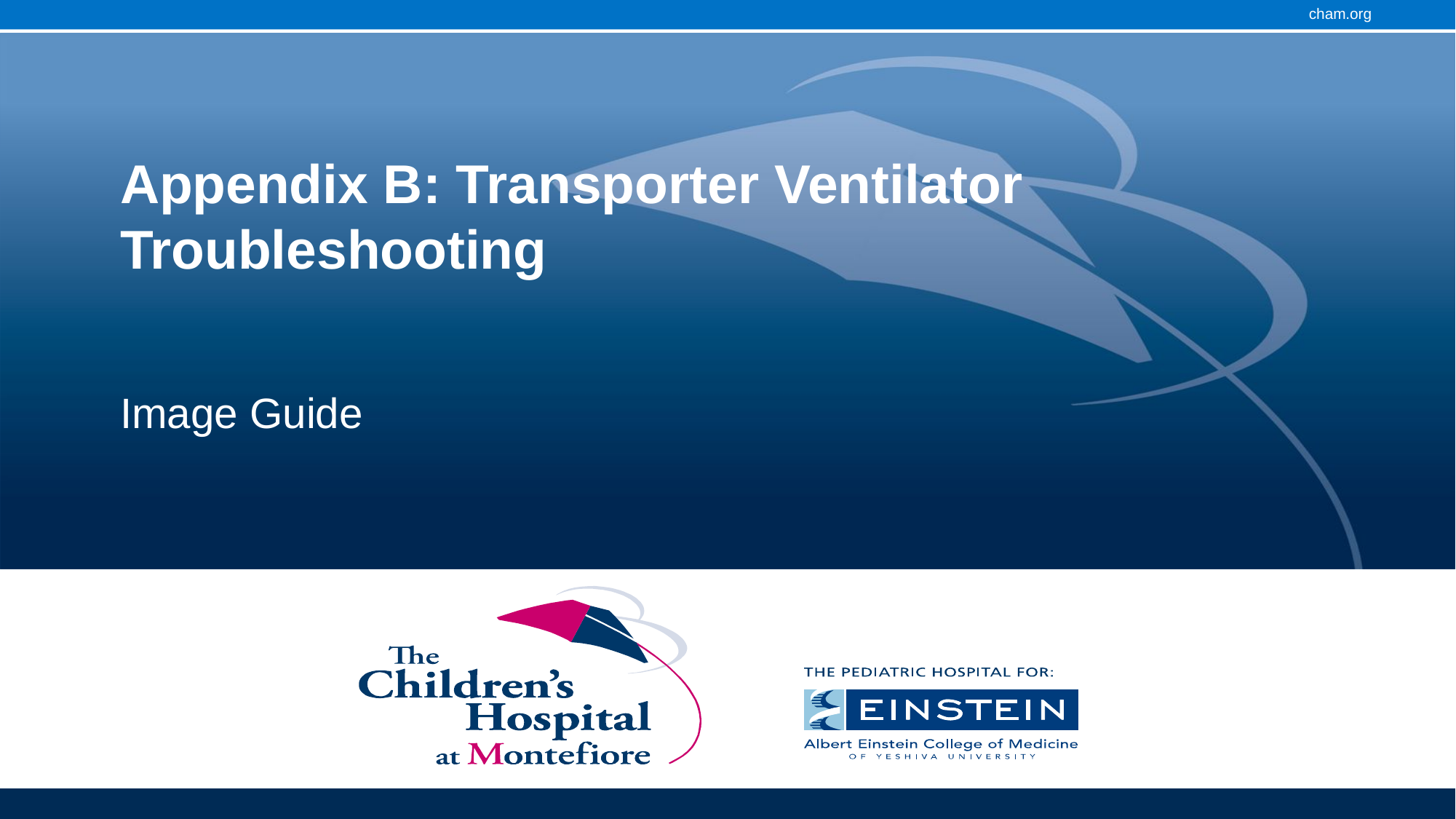

# Appendix B: Transporter Ventilator Troubleshooting
Image Guide

## Slide 2
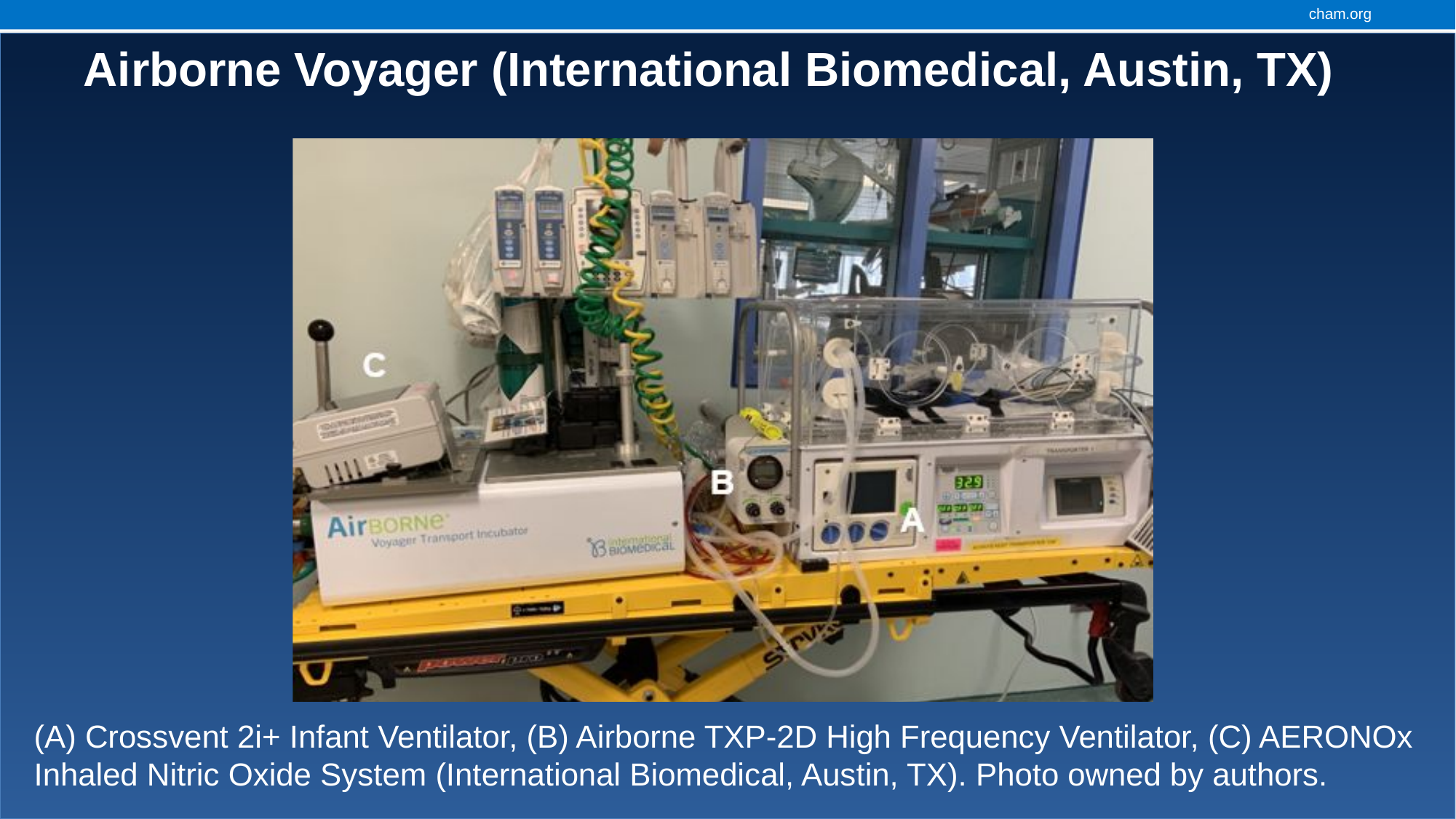

# Airborne Voyager (International Biomedical, Austin, TX)
(A) Crossvent 2i+ Infant Ventilator, (B) Airborne TXP-2D High Frequency Ventilator, (C) AERONOx Inhaled Nitric Oxide System (International Biomedical, Austin, TX). Photo owned by authors.

## Slide 3
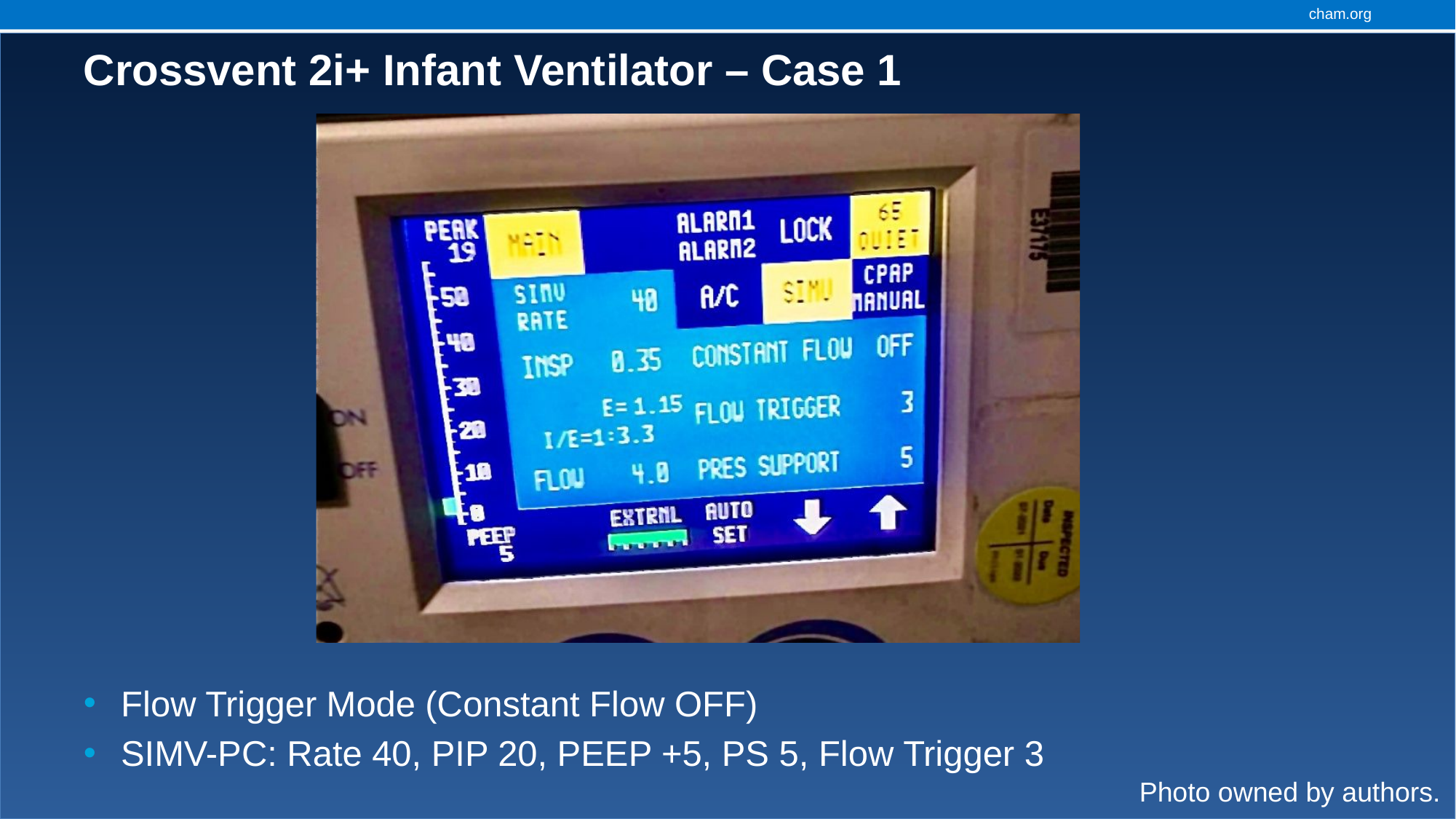

# Crossvent 2i+ Infant Ventilator – Case 1
Flow Trigger Mode (Constant Flow OFF)
SIMV-PC: Rate 40, PIP 20, PEEP +5, PS 5, Flow Trigger 3
Photo owned by authors.

## Slide 4
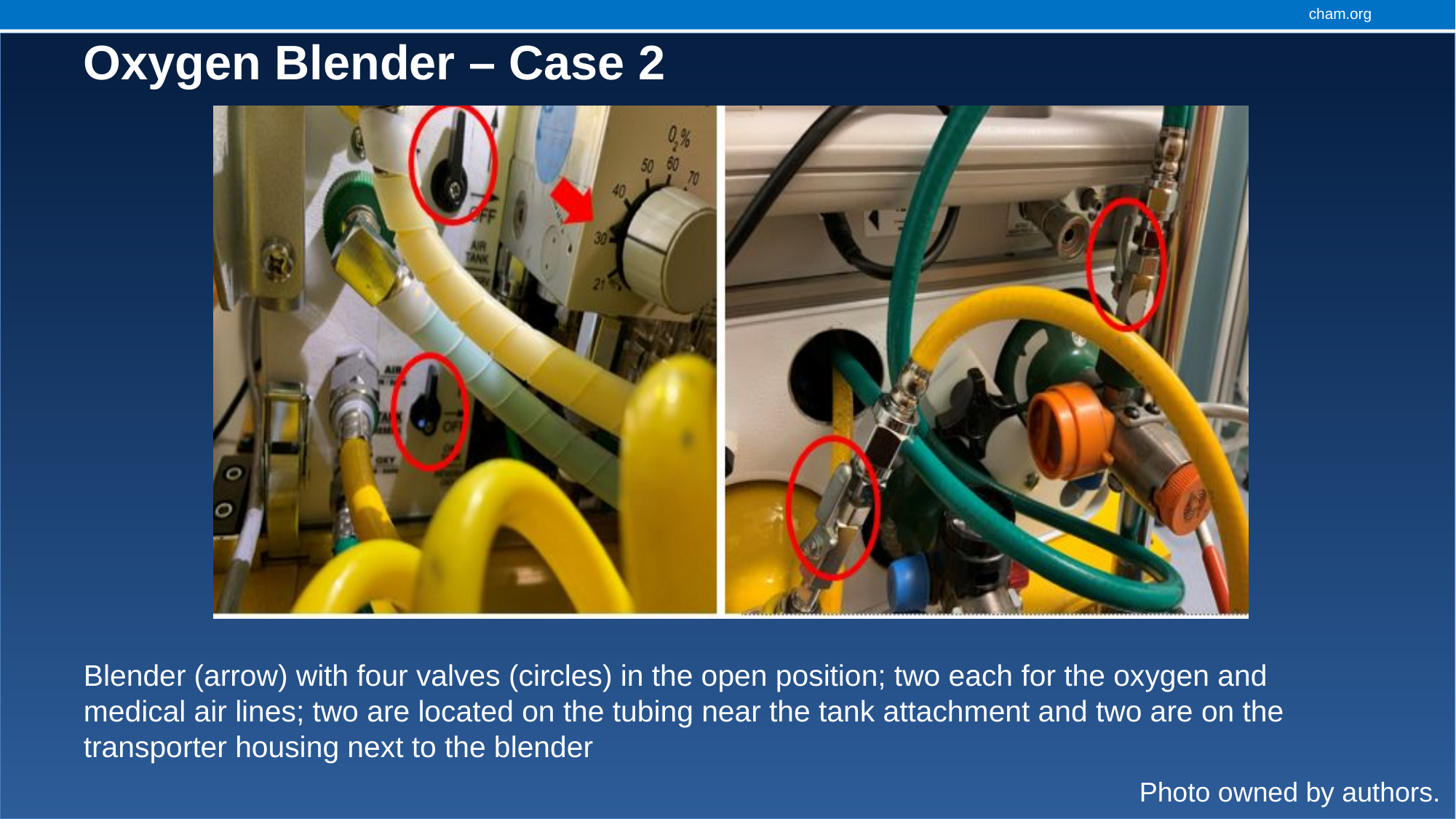

# Oxygen Blender – Case 2
Blender (arrow) with four valves (circles) in the open position; two each for the oxygen and medical air lines; two are located on the tubing near the tank attachment and two are on the transporter housing next to the blender
Photo owned by authors.

## Slide 5
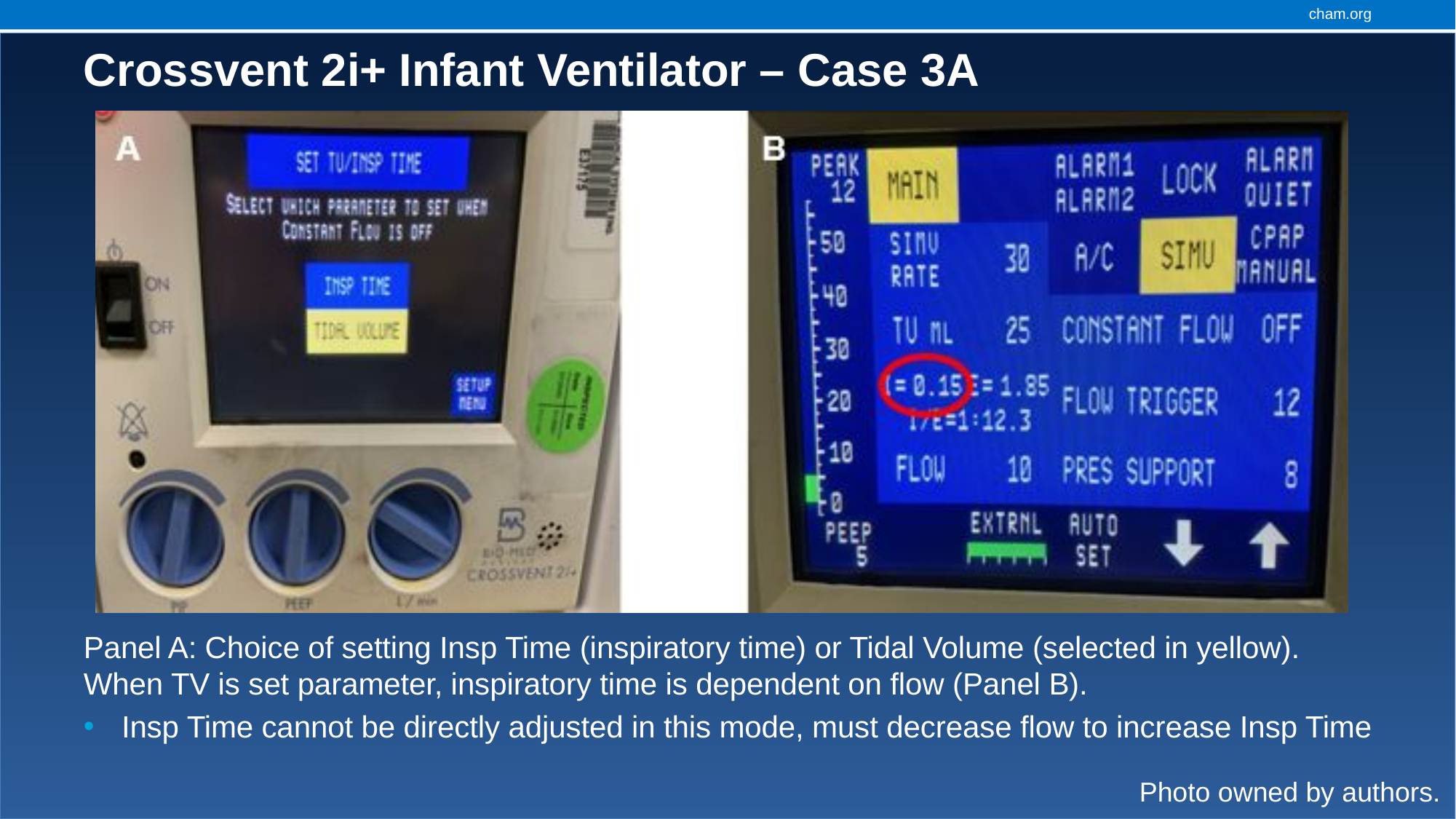

# Crossvent 2i+ Infant Ventilator – Case 3A
Panel A: Choice of setting Insp Time (inspiratory time) or Tidal Volume (selected in yellow). When TV is set parameter, inspiratory time is dependent on flow (Panel B).
Insp Time cannot be directly adjusted in this mode, must decrease flow to increase Insp Time
Photo owned by authors.

## Slide 6
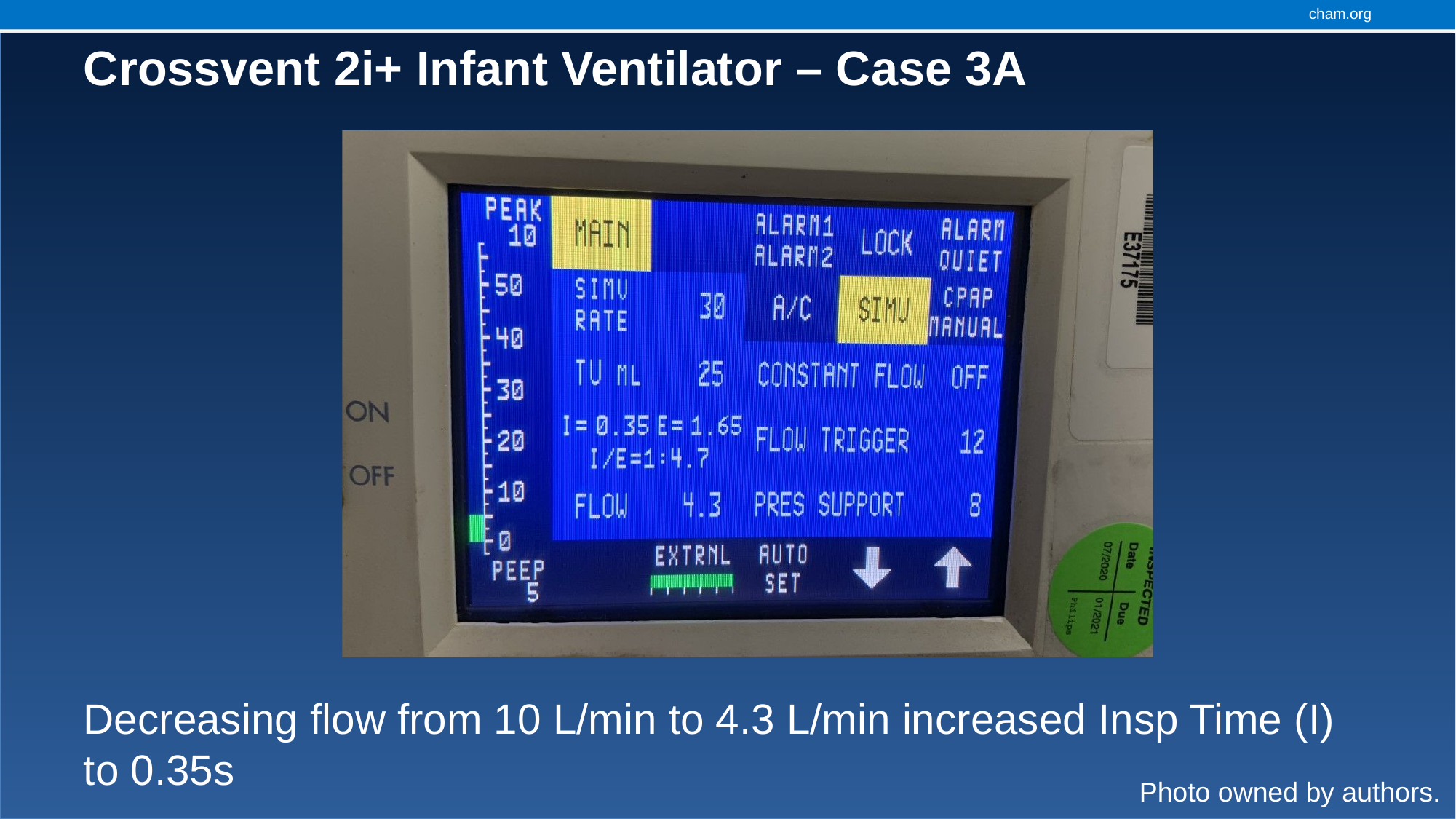

# Crossvent 2i+ Infant Ventilator – Case 3A
Decreasing flow from 10 L/min to 4.3 L/min increased Insp Time (I) to 0.35s
Photo owned by authors.

## Slide 7
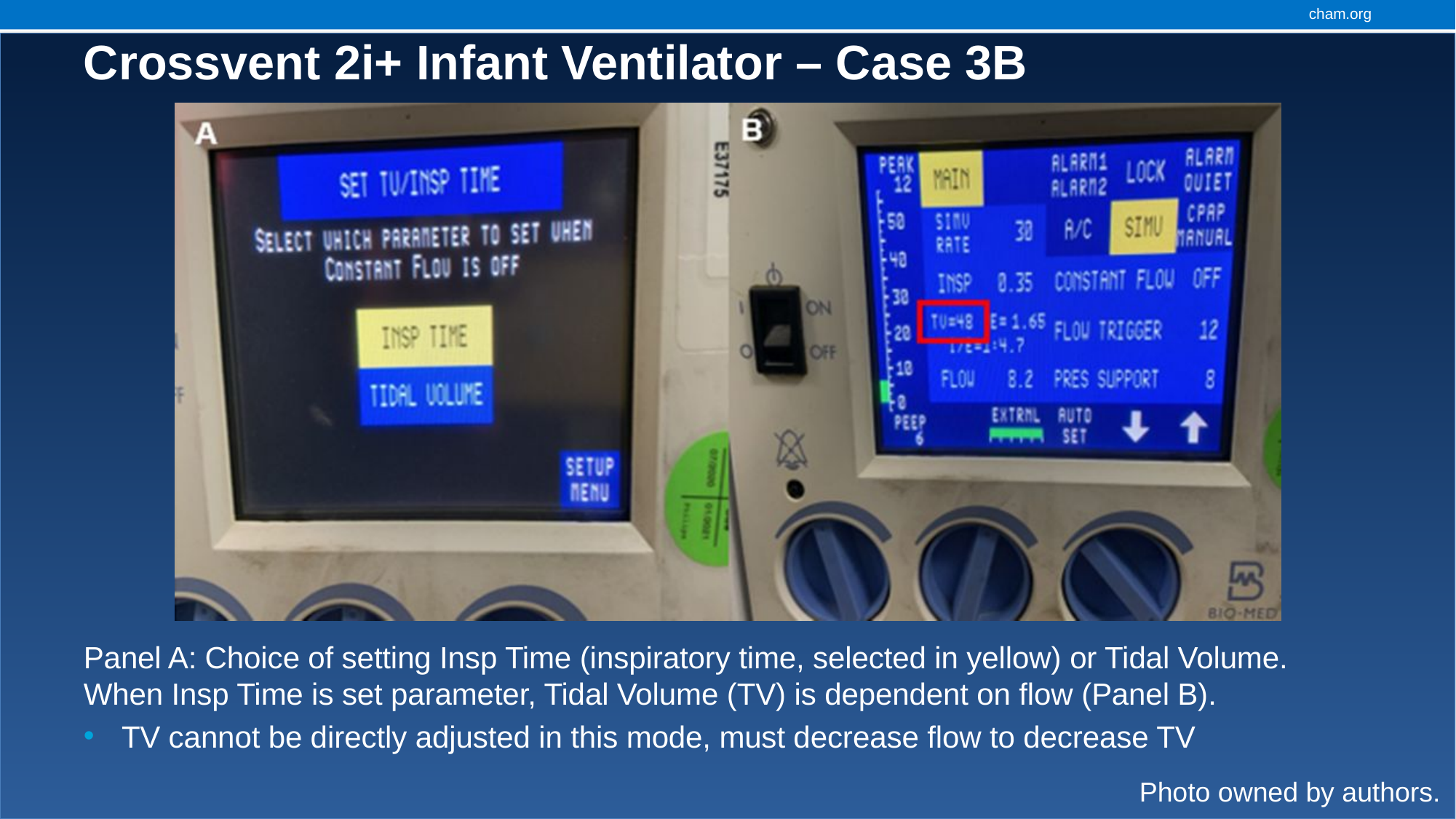

# Crossvent 2i+ Infant Ventilator – Case 3B
Panel A: Choice of setting Insp Time (inspiratory time, selected in yellow) or Tidal Volume. When Insp Time is set parameter, Tidal Volume (TV) is dependent on flow (Panel B).
TV cannot be directly adjusted in this mode, must decrease flow to decrease TV
Photo owned by authors.

## Slide 8
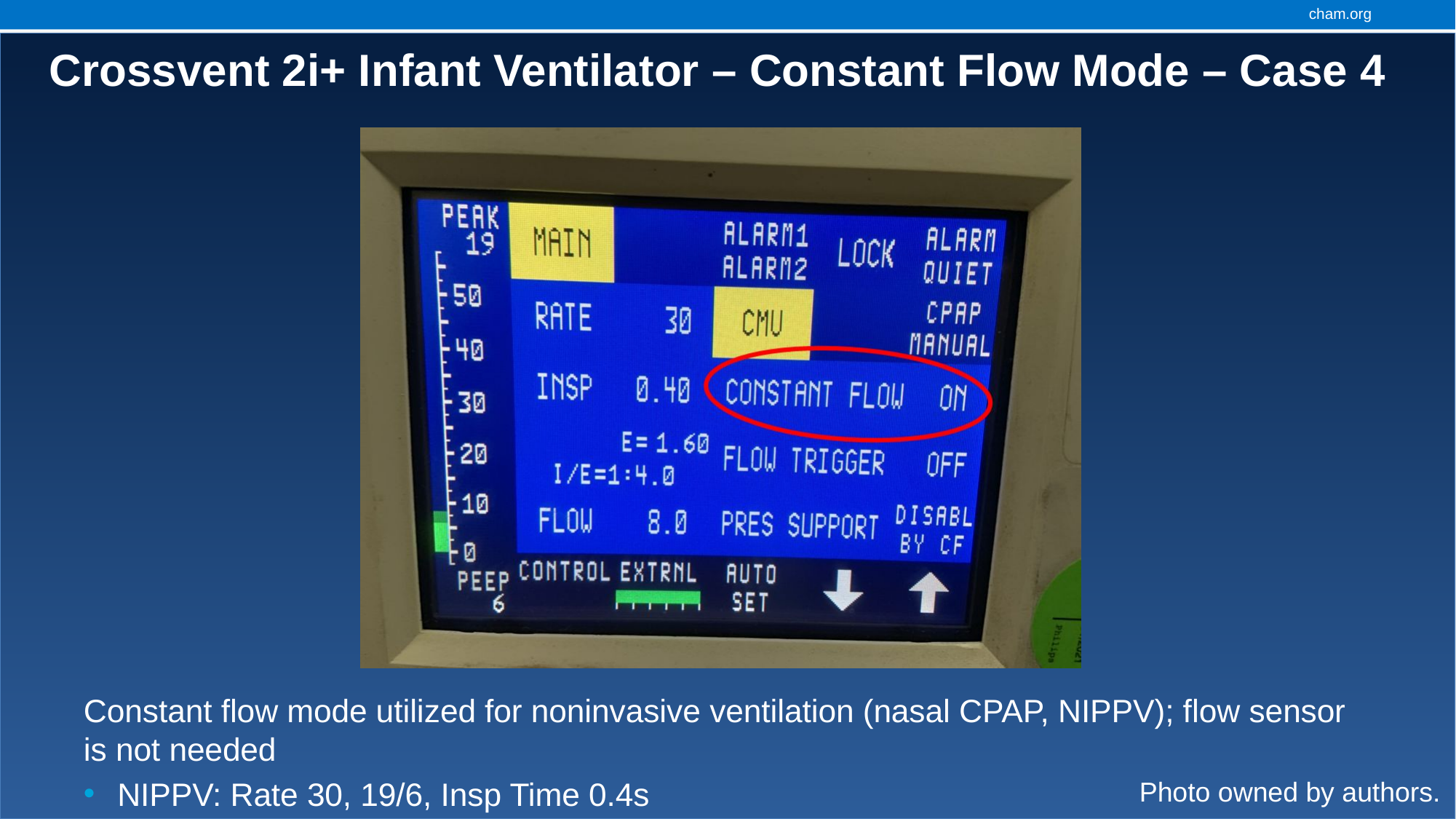

# Crossvent 2i+ Infant Ventilator – Constant Flow Mode – Case 4
Constant flow mode utilized for noninvasive ventilation (nasal CPAP, NIPPV); flow sensor is not needed
NIPPV: Rate 30, 19/6, Insp Time 0.4s
Photo owned by authors.

## Slide 9
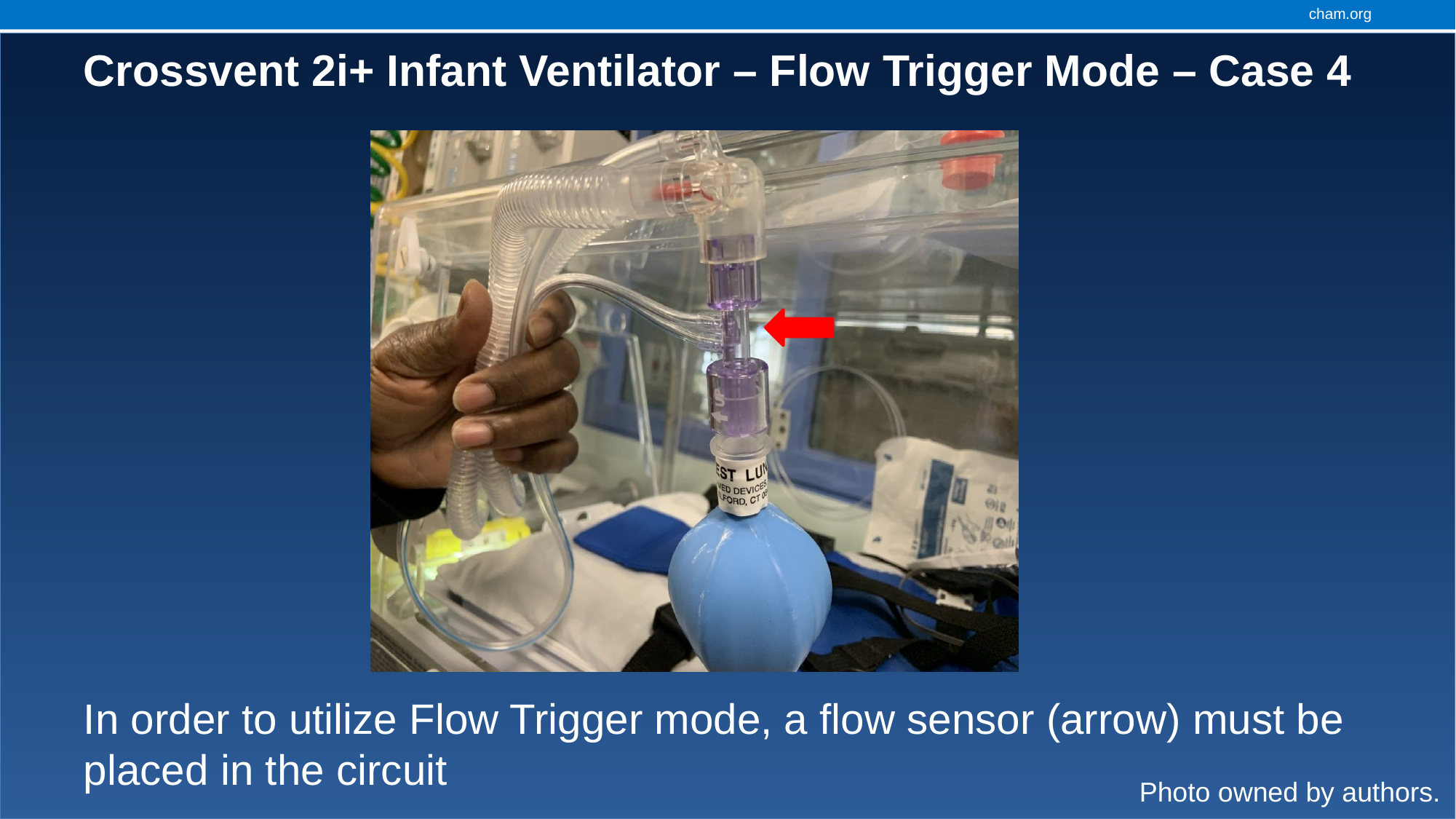

# Crossvent 2i+ Infant Ventilator – Flow Trigger Mode – Case 4
In order to utilize Flow Trigger mode, a flow sensor (arrow) must be placed in the circuit
Photo owned by authors.

## Slide 10
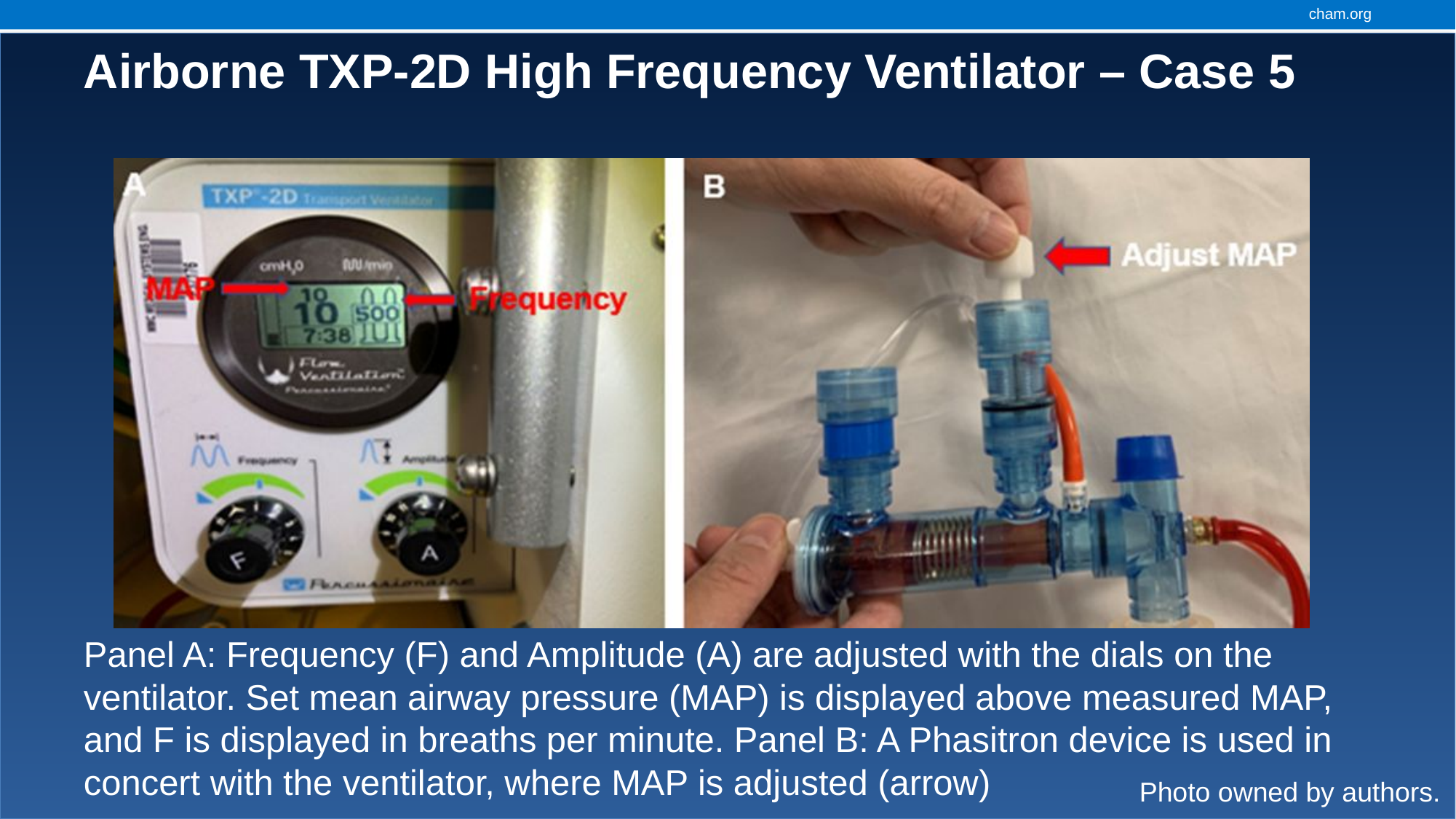

# Airborne TXP-2D High Frequency Ventilator – Case 5
Panel A: Frequency (F) and Amplitude (A) are adjusted with the dials on the ventilator. Set mean airway pressure (MAP) is displayed above measured MAP, and F is displayed in breaths per minute. Panel B: A Phasitron device is used in concert with the ventilator, where MAP is adjusted (arrow)
Photo owned by authors.

## Slide 11
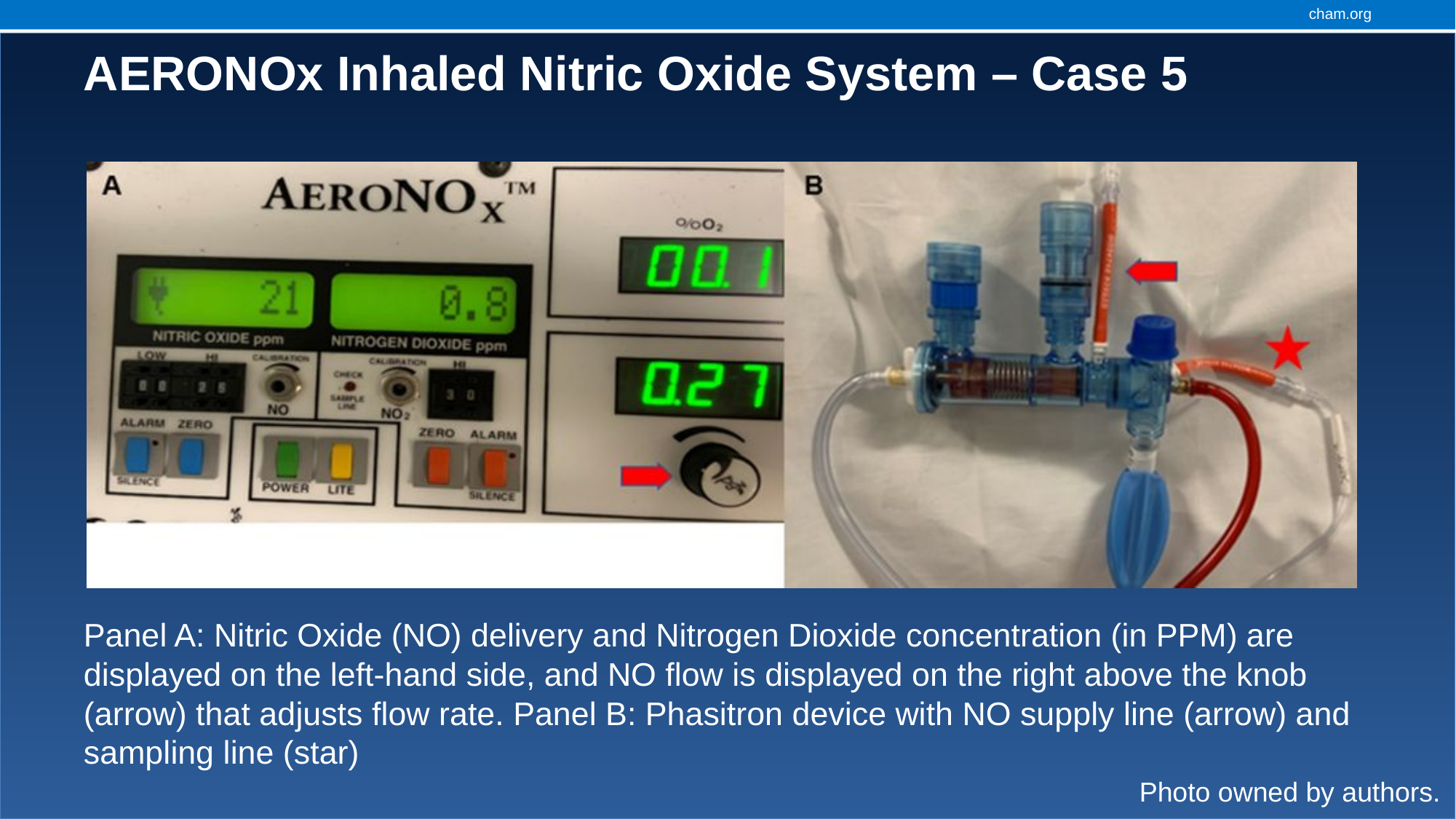

# AERONOx Inhaled Nitric Oxide System – Case 5
Panel A: Nitric Oxide (NO) delivery and Nitrogen Dioxide concentration (in PPM) are displayed on the left-hand side, and NO flow is displayed on the right above the knob (arrow) that adjusts flow rate. Panel B: Phasitron device with NO supply line (arrow) and sampling line (star)
Photo owned by authors.
