# Supplementary material for: Neonatal Transport Ventilation: Simulation to Improve Knowledge and Skills
Source: MedEdPORTAL. 2022 Sep 13;18:11272. doi: 10.15766/mep_2374-8265.11272 (PMC9468152; doi:10.15766/mep_2374-8265.11272)
Supplement: Supplementary file 1 — Simulation Scenarios Guide.docxTransport Ventilator Troubleshooting Visual Aid.pptxPostsession Survey.docxLearner Knowledge Test.docxKnowledge Test Answers.docx [file mep_2374-8265.11272-s001.zip › A. Simulation Scenarios Guide.docx]

| **Appendix A: MedEdPORTAL Simulation Case 1**  **SIMULATION CASE TITLE: Neonatal Transport Ventilation: Simulation to Improve Knowledge and Skills**  **Intubated Preterm Infant with Respiratory Distress Syndrome**  **AUTHORS: Orna Rosen, MD**  **LEARNER AUDIENCE: Neonatal-perinatal fellows, nurse practitioners, physician assistants and respiratory therapists** | |
| --- | --- |
| **PATIENT AGE: 0-day old neonate born at 27 weeks’ gestation** | |
|  | |
| **Brief narrative description of case** | A male infant born at 27 weeks’ gestation weighing 900 grams is intubated with 2.5mm endotracheal tube due to respiratory distress syndrome. He requires transport to your NICU.  Learners are expected to identify and correct auto-cycling and loss of peak inspiratory pressure (PIP) when ventilating an intubated infant. |
| **Primary Learning Objectives** | 1. Recognize that the administered respiratory rate is higher than the set respiratory rate  2. List potential causes of auto-cycling  3. Specify which of the potential causes is resulting in auto-cycling  4. Correct the auto-cycling by increasing the ventilator’s flow trigger  5. Recognize Loss of PIP alarm in setting of dropping oxygen saturations  6. List potential causes of loss of PIP  7. Prepare to use back-up ventilation device (positive pressure ventilation (PPV) via T-piece resuscitator or self-inflating bag) if unable to identify cause of loss of PIP |
| **Critical Actions** | 1. Assess whether the ventilator is providing the intended support  2. Check for air leak within circuit  3. Confirm endotracheal tube position  4. Determine that auto-cycling is due to ambulance and transport incubator movement (bumpy road)  5. Increase flow trigger to eliminate auto-cycling  6. Check air and oxygen tanks to ensure they are not empty  7. Utilize alternate form of PPV while troubleshooting or if unable to identify cause while neonate is decompensating |
| **Learner Preparation or Prework** | 19-item knowledge test (Appendix D) |

| Initial Presentation | |
| --- | --- |
| **Initial vital signs** | Weight 900g Respiratory Rate 60 Oxygen Saturation 92% |
| **Overall Setting and Appearance** | NICU Transport Ventilator is set up and delivering support to an artificial lung (Appendix B, Slide 3)  Vent Settings:  Flow Trigger 3  Rate 40  PIP 20 cmH2O  Positive End Expiratory Pressure (PEEP) 5 cmH2O  Pressure Support 5 cmH2O  Fraction of Inspired Oxygen (FiO2) 30% |
| **Standardized Participants (and their roles in the room at case start**) | None |
| **HPI** | A male infant born at 27 weeks’ gestation due preterm labor. He is orally intubated with a 2.5mm endotracheal tube to a depth of 7cm due to respiratory distress syndrome. He requires transport to a higher level NICU. |
| **Physical Examination:** (Information provided if learners ask) Intubated, mild retractions, symmetric chest rise, clear equal breath sounds, pink, well-perfused | |

| Instructor Notes - Changes and CASE Branch Points | | |
| --- | --- | --- |
| **Intervention / Time point** | **Change in Case** | **Additional Information** |
| Beginning of Case – Ventilator providing breaths at 60 per minute (above set of 40) |  | RRT manipulates the artificial lung to mimic autocycling/respiratory rate higher than ventilator rate. Road is very bumpy.  Facilitator may prompt learner if they do not identify auto-cycling |
| Vent circuit checked | No leak identified; auto-cycling continues |  |
| Endotracheal tube position is assessed | Tube inserted to 7cm, symmetric breath sounds auscultated, auto-cycling continues | Learner must ask as there’s no manikin to assess directly |
| Auto-cycling Identified due to bumpy road, Flow Trigger increased to 5 | Auto-cycling stops, delivered respiratory rate is now 40 |  |
| 1 minute later: Facilitator announces the now has an oxygen saturation of 70% and appears cyanotic |  | Senior RRT makes a leak in the circuit resulting in Low PIP reading |
| Vent circuit checked | No leak identified |  |
| Endotracheal tube position is assessed | Tube inserted to 7cm, symmetric breath sounds auscultated | No cause identified for loss of PIP |
| PPV initiated with alternate device | Saturations and cyanosis improve |  |

**Ideal Scenario Flow**

At the beginning of the scenario, the learner should compare the reported ventilator settings to what is actually being delivered. They should recognize that the delivered ventilator rate is higher than the set rate (Appendix B, Slide 3). Possible causes for this auto-cycling include a leak in the ventilator circuit, malpositioned or dislodged endotracheal tube and interference from incubator and ambulance movement from bumps in the road. The learner should troubleshoot all three possible causes and correct the auto-cycling by increasing the flow trigger to overcome the interference from ambulance movement. Shortly thereafter, the facilitator announces that the neonate has started to desaturate and appears cyanotic, and the senior RRT creates a leak in the circuit such that the delivered PIP is lower than the set PIP on the ventilator screen. The learner must recognize this loss of PIP. Possible causes for this include a leak in the ventilator circuit, malpositioned or dislodged endotracheal tube and empty air and/or oxygen tanks. The learner should troubleshoot all three possibilities. In this case, no reason for loss of PIP is identified, and the neonate remains cyanotic. The learner must utilize an alternate PPV device (T-piece resuscitator or self-inflating bag) to stabilize the infant.

**Anticipated Management Mistakes**

1. Auto-cycling is not identified: We built a facilitator prompt into the scenario if the learners fail to recognize the auto-cycling.

2. Unfamiliarity with ventilator circuit: In order to troubleshoot for leaks in the ventilator circuit, the learner needs to be familiar with the circuit and its connections. We discuss this specifically during the debrief.

**Debriefing Guide**

- Ask the learners to summarize the case
- Specifically ask the learners to identify what was malfunctioning (1. Flow trigger set too low resulting in auto-triggering on a bumpy road; 2. Unexplained loss of PIP resulting in inadequate ventilation and oxygenation of the patient)
- Highlight a systematic approach to troubleshooting a malfunctioning ventilator
  - Check the ventilator to ensure that prescribed parameters are actually being delivered to the patient
    - If the ventilator is not delivering the expected settings, ie the delivered rate is higher than the set rate, ask the learners how to make adjustments on the ventilator
  - Assess the “patient” – the learners must ask the facilitator about exam findings, ie endotracheal tube in place, symmetric breath sounds, etc
  - Check the ventilator circuit to make sure there are no leaks or disconnections
  - Check the medical air and oxygen tanks to ensure adequate gas supply
- Highlight the importance of having a back-up method to perform PPV if malfunctioning ventilator is unable to be remedied
- Ask the learners for any other comments or questions

| **Appendix A: MedEdPORTAL Simulation Case 2**  **SIMULATION CASE TITLE: Neonatal Transport Ventilation: Simulation to Improve Knowledge and Skills**  **Full Term Infant with Meconium Aspiration Syndrome – Empty Tanks**  **AUTHORS: Orna Rosen, MD**  **LEARNER AUDIENCE: Neonatal-perinatal fellows, nurse practitioners, physician assistants and respiratory therapists** | |
| --- | --- |
| **PATIENT AGE: 1-day old neonate born at 41 weeks’ gestation** | |
|  | |
| **Brief narrative description of case** | A postterm infant with meconium aspiration syndrome (MAS) is critically ill and requires transfer from a referring institution. She is intubated with a 3.5 endotracheal tube and is on pressure control ventilation. The transport is uneventful, but on exiting the ambulance upon arrival back to the hospital, a loud, high-pitched noise is heard.  Learners are expected to be familiar with the gas tank hook-ups, identify alarms and other troubling sounds the transport ventilator can make and rectify the situation. |
| **Primary Learning Objectives** | 1. Recognize that the high-pitched noise is originating from the oxygen blender  2. Identify that all four valves (two each leading from the oxygen and medical air tanks) are in the correct and open position  3. Identify that one or both tanks are empty.  4. Perform the corrective action of changing out the empty tank  5. Recognize that if the medical air tank is empty and/or there isn’t a replacement, the medical air line can be shut off, and the neonate may be briefly placed on 100% oxygen for the last leg of the transport. |
| **Critical Actions** | 1. Assess that the ventilator is delivering the intended support  2. Appreciate that the high-pitched noise is originating from the blender  3. Check to ensure that all four valves are in the open position (Appendix B, Slide 4)  4. Check the medical air and oxygen tanks to see if either are empty  5. Identify which is empty and replace with a back-up tank  6. If the medical air tank is empty, consider turning off the valves to the medical air line and briefly increasing to 100% FiO2 for the remainder of the transport from the ambulance to the NICU |
| **Learner Preparation or Prework** | 19-item knowledge test (Appendix D) |

| Initial Presentation | |
| --- | --- |
| **Initial vital signs** | Weight 4kg Oxygen Saturation 90% |
| **Overall Setting and Appearance** | NICU Transport Ventilator is set up and delivering support to an artificial lung  Vent Settings:  Rate 40  PIP 25 cmH2O  PEEP 5 cmH2O  Pressure Support 5 cmH2O  FiO2 50% |
| **Standardized Participants (and their roles in the room at case start**) | None |
| **HPI** | A female infant was born at 41 weeks’ gestation via urgent C-section. Her mother was undergoing an induction of labor, and labor course was complicated by category 2 fetal heart tracing and particulate meconium-stained amniotic fluid noted at time of membrane rupture. At delivery, the neonate required intubation for poor respiratory effort and cyanosis. Her FiO2 requirement has been climbing over the last 6 hours, and she now requires transport to a higher level NICU. |
| **Physical Examination:** (Information provided if learners ask) Intubated, moderate retractions, symmetric chest rise, coarse but symmetric breath sounds | |

| Instructor Notes - Changes and CASE Branch Points | | |
| --- | --- | --- |
| **Intervention / Time point** | **Change in Case** | **Additional Information** |
| Beginning of Case – In ambulance en route back to hospital |  |  |
| Upon arrival at home institution, air and oxygen source changed from ambulance to transporter. | A loud, high-pitched noise is heard when entering the elevator on the way to the NICU. | Senior RRT preferably should use an empty tank to create the noise, but may also choose to close one of the valves  Facilitator may prompt learners if they do not identify source of noise |
| All four valves are checked and found to be in the open position | Neonate begins to desaturate, and pulse oximeter is alarming low | Facilitator must prompt learners at this step |
| Oxygen tank is identified as empty | Oxygen tank is switched over to the back-up tank |  |
| The high-pitched noise is no longer heard | Cyanosis and oxygen saturations improve | Facilitator describes improved status at this step |
| **Alternate Branch Point:** Instead of empty oxygen tank, the medical air tank is identified as empty | Medical air tank is switched over to the back-up tank **OR** medical air line is closed (two valves in off position) and briefly administer 100% oxygen for the short ride up to the NICU |  |

**Ideal Scenario Flow**

At the beginning of the scenario, transport from the referring institution is in progress and uneventful. The ventilator is delivering the intended settings, and the neonate remains stable with oxygen saturation in the low 90s on 50% FiO2. Upon arrival back to the hospital, the gas source is changed from the ambulance to the transporter. Upon entering the elevator on the way to the NICU, a loud, high-pitched noise is heard. The RRT may use either an empty tank or close one of the valves to mimic this noise. The learners must identify the source of this noise (the blender), check to ensure all valves are open (Appendix B, Slide 4), look for empty oxygen and/or medical air tanks and rectify the situation by replacing the empty tank. The facilitator may choose whether to do the empty oxygen tank scenario, empty medical air tank scenario or both. If the empty medical air tank scenario is chosen, the learners may elect to administer 100% FiO2 briefly until arrival into the NICU, but this may not be ideal as even a few short minutes may expose the neonate to hyperoxia.

**Anticipated Management Mistakes**

1. Source of high-pitched noise is not identified: We built a facilitator prompt into the scenario if the learners fail to recognize the sound.

2. Unfamiliarity with tank circuits: If learners are unfamiliar with the equipment, they may not know the correct position of the tank valves or how to replace an empty tank. The purpose of this scenario is to become more familiar with the equipment, and we discuss this specifically during the debrief.

**Debriefing Guide**

- Ask the learners to summarize the case
- Specifically ask the learners to identify what was malfunctioning (Medical air and/or oxygen tank is empty), highlighting the characteristic loud whistle the blender makes when one or both of the gas tanks is/are empty
- Highlight a systematic approach to troubleshooting a malfunctioning ventilator
  - Check the medical air and oxygen tanks to ensure valves are open and delivering adequate gas supply
    - Ask or have the learners demonstrate proper, open valve position and how to replace an empty gas tank
  - Check the ventilator to ensure that prescribed parameters are actually being delivered to the patient
  - Assess the “patient” – the learners must ask the facilitator about exam findings, ie endotracheal tube in place, symmetric breath sounds, etc
  - Check the ventilator circuit to make sure there are no leaks or disconnections
- Highlight the importance of having back-up gas tanks available and utilizing the oxygen and medical air supplied by the ambulance during transport so that the tanks do not run out
- Ask the learners for any other comments or questions

| **Appendix A: MedEdPORTAL Simulation Case 3**  **SIMULATION CASE TITLE: Neonatal Transport Ventilation: Simulation to Improve Knowledge and Skills**  **Full Term Infant with Meconium Aspiration Syndrome – Ventilator Flow**  **AUTHORS: Orna Rosen, MD**  **LEARNER AUDIENCE: Neonatal-perinatal fellows, nurse practitioners, physician assistants and respiratory therapists** | |
| --- | --- |
| **PATIENT AGE: 0-day old neonate born at 40 weeks’ gestation** | |
|  | |
| **Brief narrative description of case** | A critically ill full-term neonate has been diagnosed with MAS at a referring institution and requires transport for a higher level of care. He is intubated with a 3.5 endotracheal tube and is on a conventional ventilator.  Learners should recognize that with the Crossvent 2i+ Infant Ventilator, inspiratory time and tidal volume are dependent on flow. |
| **Primary Learning Objectives** | 1. List the two modes of flow trigger ventilation on the Crossvent 2i+ Infant Ventilator (Tidal Volume and Inspiratory Time)  2. Describe the set parameters in each mode  3. Demonstrate how to set up ventilator and adjust parameters  4. Assess the “non-set” parameters once ventilation begins (assess inspiratory time in Tidal Volume mode; assess tidal volume in Inspiratory Time mode)  5. Recognize that in Tidal Volume mode, inspiratory time cannot be directly adjusted; flow must be changed  6. Recognize that in Inspiratory Time mode, tidal volume cannot be directly adjusted; flow must be changed |
| **Critical Actions** | 1. Set up Tidal Volume mode  2. Check inspiratory time (non-set parameter) while ventilating in Tidal Volume mode  3. Decrease flow (dial on right below ventilator screen) to achieve desired inspiratory time.  3. Set up Inspiratory Time mode  4. Check tidal volume (non-set parameter) while ventilating in Inspiratory Time mode  5. Decrease flow to achieve a tidal volume that will minimize volutrauma while maintaining oxygenation and ventilation |
| **Learner Preparation or Prework** | 19-item knowledge test (Appendix D) |

| Initial Presentation | |
| --- | --- |
| **Initial vital signs** | Weight 3.5kg Oxygen Saturation 94% |
| **Overall Setting and Appearance** | NICU Transport Ventilator circuit is connected to an artificial lung. The ventilator has not yet been set up.  Vignette A:  Flow Trigger  Tidal Volume Mode (Appendix B, Slide 5)  Rate 30  Tidal Volume 25ml  PEEP 5 cmH2O  Pressure Support 8 cmH2O  FiO2 40%  Vignette B:  Flow Trigger  Insp Time Mode (Appendix B, Slide 7)  Rate 30  PIP 24 cmH2O  PEEP 5 cmH2O  Pressure Support 8 cmH2O  Insp Time 0.35 second  FiO2 40% |
| **Standardized Participants (and their roles in the room at case start**) | None |
| **HPI** | A male infant was born at 39 weeks’ gestation via vaginal delivery. Labor course was complicated by category 2 fetal heart tracing and thick meconium-stained amniotic fluid noted at time of membrane rupture (12 hours prior to delivery). At delivery, the neonate required intubation for poor respiratory effort and cyanosis. His FiO2 requirement has been climbing over the last 6 hours, and he now requires transport to a higher level NICU. |
| **Physical Examination:** (Information provided if learners ask) Intubated, mild subcostal retractions, symmetric chest rise, coarse but symmetric breath sounds | |

| Instructor Notes - Changes and CASE Branch Points | | |
| --- | --- | --- |
| **Intervention / Time point** | **Change in Case** | **Additional Information** |
| **Vignette A:** |  |  |
| Ventilator set to Tidal Volume mode and parameters described above are set |  |  |
| Inspiratory Time (“non-set” parameter) is noted to be 0.15 seconds at a flow of 10L per minute |  | Learners must assess that this is an inadequate inspiratory time. Facilitator may prompt learners to assess inspiratory time while ventilating in Tidal Volume mode. |
| Flow rate decreased to 4.3L per minute | Inspiratory Time increase to 0.35s | Facilitator may refer to Appendix B, Slide 6 |
| **Vignette B:** |  |  |
| Ventilator set to Inspiratory Time mode and parameters described above are set |  |  |
| Tidal Volume (“non-set” parameter) is noted to be 48ml at a flow of 8.2L per minute |  | Learners must assess that this is an excessive tidal volume (Neonate is 3.5kg so that tidal volume is >13ml/kg). Facilitator may prompt learners to assess tidal volume while ventilating in Inspiratory Time mode. |
| Flow rate is decreased until a more appropriate tidal volume is achieved | Goal tidal volume is < 10ml/kg or < 35ml |  |

**Ideal Scenario Flow**

Vignette A: At the start of the scenario, the learner should set up the ventilator to the settings listed above. They should first select Tidal Volume mode, and then input the desired settings (Appendix B, Slide 5). As ventilation begins, they should assess the ventilator screen to ensure that the desired support is being administered. They should also note the Inspiratory Time and Flow. They must recognize that the Inspiratory Time is not adequate and that it cannot be directly adjusted when utilizing Tidal Volume mode. Then, the flow must be decreased until the desired Inspiratory Time is achieved (Appendix B, Slide 6).

Vignette B: At the start of the scenario, the learner should set up the ventilator to the settings listed above. They should first select Inspiratory Time mode, and then input the desired settings (Appendix B, Slide 7). As ventilation begins, they should assess the ventilator screen to ensure that the desired support is being administered. They should also note the Tidal Volume and Flow. They must recognize that the Tidal Volume is excessive and that it cannot be directly adjusted in Inspiratory Time mode. Then, the flow must be decreased until the desired Tidal Volume is achieved (< 10ml/kg).

**Anticipated Management Mistakes**

1. Unfamiliarity with Crossvent 2i+ Infant Ventilator: If learners are unfamiliar with the ventilator, they may not know how to correctly set up different modes of ventilation. The purpose of this scenario is to become more familiar with the equipment, and we discuss this specifically during the debrief.

2. Inadequate Inspiratory Time not identified: We built a facilitator prompt into the scenario if the learners fail to recognize the low inspiratory time.

3. Excessive Tidal Volume not identified: We built a facilitator prompt into the scenario if the learners fail to recognize the excessive tidal volume.

**Debriefing Guide**

- Ask the learners to summarize the case
- Specifically ask the learners to identify what was malfunctioning (A: Inappropriately low inspiratory time when utilizing Tidal Volume mode; B: Inappropriately high tidal volume when utilizing Insp Time mode)
- Highlight a systematic approach to troubleshooting a malfunctioning ventilator
  - Check the ventilator to ensure that prescribed parameters are actually being delivered to the patient
    - Ask or have the learners demonstrate how to make changes on the ventilator to remedy the identified inappropriate parameters
  - Assess the “patient” – the learners must ask the facilitator about exam findings, ie endotracheal tube in place, symmetric breath sounds, etc
  - Check the ventilator circuit to make sure there are no leaks or disconnections
  - Check the medical air and oxygen tanks to ensure valves are open and delivering adequate gas supply
- Ask the learners for any other comments or questions

| **Appendix A: MedEdPORTAL Simulation Case 4**  **SIMULATION CASE TITLE: Neonatal Transport Ventilation: Simulation to Improve Knowledge and Skills**  **Preterm Infant with Respiratory Distress Syndrome – Constant and Trigger Flow Modes**  **AUTHORS: Orna Rosen, MD**  **LEARNER AUDIENCE: Neonatal-perinatal fellows, nurse practitioners, physician assistants and respiratory therapists** | |
| --- | --- |
| **PATIENT AGE:** 0-day old neonate born at 28 weeks’ gestation | |
|  | |
| **Brief narrative description of case** | A female infant born at 28 weeks’ gestation requires non-invasive positive pressure ventilation (NIPPV) for respiratory distress syndrome. She requires transport to your NICU.  Learners are expected to understand the difference between constant flow and trigger flow modes on the Crossvent 2i+ Infant Ventilator and be able to set up both modes. |
| **Primary Learning Objectives** | 1. Describe how to set up nasal intermittent ventilation (NIV) on the Crossvent 2i+ Infant Ventilator  2. Describe how to change to an invasive ventilation mode on the Crossvent 2i+ Infant Ventilator  3. Recognize that a functioning flow sensor is required for ventilation in flow trigger mode. |
| **Critical Actions** | 1. Set up ventilator with Constant Flow (Flow Trigger Off)  2. Change to Flow Trigger (Constant Flow Off) when neonate is intubated and requires invasive ventilation  3. Change back to Constant Flow when notified by RRT that the flow sensor is cracked and non-functioning |
| **Learner Preparation or Prework** | 19-item knowledge test (Appendix D) |

| Initial Presentation | |
| --- | --- |
| **Initial vital signs** | Weight 960g Respiratory Rate 55 Oxygen Saturation 92% |
| **Overall Setting and Appearance** | NICU Transport Ventilator circuit is connected to an artificial lung. The ventilator has not yet been set up.  Vent Settings (Appendix B, Slide 8):  Constant Flow (Flow Trigger Off)  Rate 30  PIP 19 cmH2O  PEEP 6 cmH2O  Inspiratory Time 0.4 seconds  FiO2 35% |
| **Standardized Participants (and their roles in the room at case start**) | None |
| **HPI** | A female infant was born at 28 weeks’ gestation via C-section due to severe, worsening maternal preeclampsia. Mother received one dose of betamethasone approximately 10 hours prior to delivery and was started on magnesium before C-section. At delivery, neonate was started on nasal CPAP and has been escalated to NIPPV due to occasional apneic events. She requires transfer to a higher level NICU. |
| **Physical Examination:** (Information provided if learners ask) Appropriate for gestational age preterm infant with large nasal prongs in place, moderate subcostal retractions, symmetric chest rise, coarse but symmetric breath sounds, pink, warm, well-perfused | |

| Instructor Notes - Changes and CASE Branch Points | | |
| --- | --- | --- |
| **Intervention / Time point** | **Change in Case** | **Additional Information** |
| At beginning of scenario, learners set up ventilator to administer NIPPV according to above settings |  | Facilitator may prompt learners to turn on Constant Flow and select CMV (conventional mandatory ventilation) to begin NIPPV |
| Facilitator reports the neonate is intubated due to recurrent apneas | Change to Flow Trigger and add Flow Sensor to circuit |  |
| RRT notifies learners that there’s a crack in the flow sensor and it’s no longer functioning | Change back to Constant Flow | Facilitator may refer to Appendix B, Slide 9 |

**Ideal Scenario Flow**

At the start of the scenario, the learner should set up the ventilator to the settings listed above. They should choose Constant Flow to administer nasal support (Appendix B, Slide 8). CMV is selected to administer NIPPV; continuous positive airway pressure (CPAP) is the other constant flow option. Once the ventilator is set up, the learners are notified of a change in patient status, that the neonate has now been intubated. They much change to Flow Trigger on the ventilator and add a flow sensor to the ventilator circuit. As they begin to set up the ventilator for invasive ventilation, the RRT should alert the learners to the non-functioning flow sensor (Appendix B, Slide 9). In light of this, they must choose to switch back to Constant Flow.

**Anticipated Management Mistakes**

1. Unfamiliarity with Crossvent 2i+ Infant Ventilator: If learners are unfamiliar with the ventilator, they may not know how to correctly set up different modes of ventilation. The purpose of this scenario is to become more familiar with the equipment, and we discuss this specifically during the debrief.

2. Unfamiliarity with ventilator circuit: To use the flow sensor, the learner needs to be familiar with the circuit and how the flow sensor attaches. We discuss this specifically during the debrief.

**Debriefing Guide**

- Ask the learners to summarize the case
- Specifically ask the learners to identify what needed to be troubleshooted (initially setting up the ventilator in Constant Flow mode to deliver nasal ventilator, recognizing that once the infant is intubated, Flow Trigger mode should be utilized)
  - Ask or have the learners demonstrate setting up nasal ventilation (Constant Flow mode) and invasive ventilation (Flow Trigger mode)
- Highlight a systematic approach to troubleshooting a malfunctioning ventilator
  - Check the ventilator to ensure that prescribed parameters are actually being delivered to the patient
  - Assess the “patient” – the learners must ask the facilitator about exam findings, ie endotracheal tube in place, symmetric breath sounds, etc
  - Check the ventilator circuit to make sure there are no leaks or disconnections
  - Check the medical air and oxygen tanks to ensure valves are open and delivering adequate gas supply
- Highlight the importance of back-up modes of ventilation that can be used in the event of damaged or missing equipment (ie flow sensor is broken, but CMV in Constant Flow mode can still be used)
  - Reinforce that CMV in Constant Flow mode is not synchronized to patient effort
- Ask the learners for any other comments or questions

| **Appendix A: MedEdPORTAL Simulation Case 5**  **SIMULATION CASE TITLE: Neonatal Transport Ventilation: Simulation to Improve Knowledge and Skills**  **Full Term Infant with Meconium Aspiration Syndrome and Pulmonary Hypertension**  **AUTHORS: Orna Rosen, MD**  **LEARNER AUDIENCE: Neonatal-perinatal fellows, nurse practitioners, physician assistants and respiratory therapists** | |
| --- | --- |
| **PATIENT AGE: 2-day old neonate born at 40 weeks’ gestation** | |
|  | |
| **Brief narrative description of case** | 2do term male infant with MAS is intubated with a 3.5 endotracheal tube on high frequency oscillatory ventilation and inhaled nitric oxide. He requires transport to your NICU.  Learners are expected to be familiar with the high frequency transport ventilator (Airborne TXP-2D High Frequency Ventilator) and nitric oxide delivery system (AERONOx Inhaled Nitric Oxide System (iNO)) in order to transport a critically ill neonate. |
| **Primary Learning Objectives** | 1. Describe how to set up the high frequency (HF) transport ventilator and how to adjust frequency, mean airway pressure and amplitude  2. Recall that adjusting one HF parameter may alter the others (including flow of iNO) and they may need to be adjusted to maintain level of support  3. Describe how to set up inhaled nitric oxide including how to correctly attach the NO supply and sampling lines  4. Identify nitrogen dioxide accumulation and describe how to bleed it out of the circuit |
| **Critical Actions** | 1. Identify dropping iNO  2. Check iNO tank to ensure not empty, make sure the valves are in open position, and tighten connections at iNO supply and sampling lines  3. Troubleshoot loss of “wiggle:” confirm endotracheal tube position, suction to relieve obstruction and check for leaks in the vent circuit  4. Assess all ventilator settings when one parameter is adjusted (i.e. if amplitude is increased, mean airway pressure (MAP), frequency and iNO must be checked and adjusted as needed to maintain support)  5. Bleed nitrogen dioxide out of circuit by briefly disconnecting the circuit from the endotracheal tube  6. Identify cause of desaturation in neonate: confirm endotracheal tube position, suction to relieve obstruction and check for leaks in the vent circuit |
| **Learner Preparation or Prework** | 19-item knowledge test (Appendix D) |

| Initial Presentation | |
| --- | --- |
| **Initial vital signs** | Weight 3.2 kg Oxygen Saturation 85% |
| **Overall Setting and Appearance** | NICU Transport Ventilator with iNO is set up and delivering support to an artificial lung  Vent Settings (Appendix B, Slide 10)  MAP 10 cmH2O  Frequency 50  FiO2 100%  iNO 20 parts per million (Appendix B, Slide 11) |
| **Standardized Participants (and their roles in the room at case start**) | None |
| **HPI** | A 2-day old male infant born at 39 weeks’ gestation via urgent C-section due to non-reassuring fetal status. He is orally intubated with a 3.5mm endotracheal tube to a depth of 9.5cm due to MAS. Due to worsening oxygenation and climbing oxygenation index at the referring institution, he was transitioned to high frequency oscillatory ventilation and was started on iNO. He requires transport to a higher level NICU for extracorporeal life support (ECMO) evaluation. |
| **Physical Examination:** (Information provided if learners ask) Intubated term infant, moderate subcostal retractions, symmetric chest rise, symmetric breath sounds, wiggling from shoulders to groin | |

| Instructor Notes - Changes and CASE Branch Points | | |
| --- | --- | --- |
| **Intervention / Time point** | **Change in Case** | **Additional Information** |
| Beginning of Case – Transport in progress. Measured iNO has dropped below 20 parts per million |  | RRT may loosen sampling line or decrease iNO flow to mimic drop in parts per million  Facilitator may prompt learners to assess iNO level if drop not identified. |
| iNO tank and valve are checked | Tank is full, valve is open. iNO remains less than 20 parts per million |  |
| iNO flow is increased | iNO remains less than 20 parts per million |  |
| iNO circuit is checked, supply and sampling lines are tightened | iNO level increases to 20 parts per million | RRT may also increase iNO flow to achieve 20 parts per million |
| 30 seconds later: Facilitator reports that the neonate is no longer “wiggling” |  |  |
| Endotracheal tube position checked, tube is suctioned | No improvement in “wiggle” |  |
| Ventilator circuit checked | No leak identified, no improvement in “wiggle” |  |
| Amplitude increased on HF transport ventilator until “wiggle” improved | MAP no longer at 10 cmH2O, iNO reading less than 20 parts per million |  |
| Identify that change in amplitude altered other settings, adjust back to above settings |  | Facilitator may prompt learners to assess other parameters if change not identified |
| 30 seconds later: RRT alerts team that nitrogen dioxide level is climbing |  |  |
| Vent circuit is briefly disconnected from endotracheal tube to bleed out nitrogen dioxide | Nitrogen dioxide level decreases |  |
| 30 seconds later: Team arrives back at home institution and is told neonate is desaturating |  |  |
| Endotracheal tube position checked, tube is suctioned | No improvement in oxygenation |  |
| Ventilator circuit checked | No improvement in oxygenation |  |
| MAP increased on HF transport ventilator until oxygenation improves | Frequency no longer at 500, iNO not reading 20 parts per million |  |
| Identify that change in MAP altered other settings, adjust back to above settings |  | Facilitator may prompt learners to assess other parameters if change not identified |

**Ideal Scenario Flow**

The scenario begins while transport is in progress. The learners should compare reported ventilator settings and iNO to what is actually being delivered (Appendix B, Slide 10). They first must address suboptimal iNO delivery demonstrated by low iNO parts per million. They should check to make sure the NO tank is full, that the valve is in the open position, and correct any leaks in the ventilator circuit, particularly at the supply and sampling line (Appendix B, Slide 11). Once they tighten the connections, the iNO will increase to 20 parts per million. If they attempt to increase iNO flow prior to tightening the connections, iNO level will not increase. Next, they must address suboptimal ventilation (facilitator describes loss of “wiggle” to the team). They should ensure that the endotracheal tube is in correct position and is patent. They should also check the circuit for leaks. No cause is identified, so they learners should increase amplitude on the HF ventilator until they achieve adequate wiggle. Any time a setting is adjusted on the Airborne TXP-2D high frequency ventilator, all other settings (MAP, frequency and iNO) must be checked and adjusted as needed to maintain support. The facilitator may prompt the learners to do this if not done spontaneously. Next, the RRT alerts the team of high nitrogen dioxide in the circuit. The learners must correct this by briefly detaching the circuit from the endotracheal tube to bleed the nitrogen dioxide out of the system. Last, the learners must address suboptimal oxygenation (facilitator describes that the neonate begins to desaturate). They should ensure that the endotracheal tube is in correct position and is patent. They should also check the circuit for leaks. No cause is identified, so they learners should increase MAP on the HF ventilator until they achieve improved oxygenation. Any time a setting is adjusted on the Airborne TXP-2D high frequency ventilator, all other settings (amplitude, frequency and iNO) must be checked and adjusted as needed. The facilitator may prompt the learners to do this if not done spontaneously.

**Anticipated Management Mistakes**

1. Unfamiliarity with Airborne TXP-2D High Frequency Ventilator: If learners are unfamiliar with the ventilator, they may not know how to correctly set it up and adjust settings. They may also not recognize that adjusting one parameter may alter the other settings (that then require additional adjustment to maintain level of support). The purpose of this scenario is to become more familiar with the equipment, and we discuss this specifically during the debrief.

2. Unfamiliarity with ventilator circuit: If learners are unfamiliar with the circuit and phasitron device, they may not know how to properly set up and use the AERONOx Inhaled Nitric Oxide System. We discuss this specifically during the debrief.

**Debriefing Guide**

- Ask the learners to summarize the case
- Specifically ask the learners to identify what was malfunctioning (1. Low iNO; 2. Inadequate ventilation of patient; 3. Elevated nitrogen dioxide; 4. Inadequate oxygenation of patient)
- Ask or have the learners demonstrate how to adjust MAP, amplitude, frequency and iNO flow
- Highlight a systematic approach to troubleshooting a malfunctioning ventilator
  - Check the ventilator to ensure that prescribed parameters are actually being delivered to the patient
    - This is of particular importance when making adjustments to high frequency ventilator settings, because adjusting one parameter (ie increasing amplitude to achieve good “wiggle,” or increasing MAP to improve oxygenation) can alter all other parameters, including iNO
  - Assess the “patient” – the learners must ask the facilitator about exam findings, ie endotracheal tube in place, symmetric breath sounds, etc
  - Check the ventilator circuit to make sure there are no leaks or disconnections
  - Check the medical air and oxygen tanks to ensure valves are open and delivering adequate gas supply
- Ask the learners for any other comments or questions
- Have the learners complete the Postsession Survey (Appendix C)
