# Supplementary material for: Neonatal Transport Ventilation: Simulation to Improve Knowledge and Skills
Source: MedEdPORTAL. 2022 Sep 13;18:11272. doi: 10.15766/mep_2374-8265.11272 (PMC9468152; doi:10.15766/mep_2374-8265.11272)
Supplement: Supplementary file 1 — Simulation Scenarios Guide.docxTransport Ventilator Troubleshooting Visual Aid.pptxPostsession Survey.docxLearner Knowledge Test.docxKnowledge Test Answers.docx [file mep_2374-8265.11272-s001.zip › C. Postsession Survey.docx]

**Appendix C:** Postsession Survey

Answer the following on a scale of 1 to 5 (1 – strongly agree; 5 – strongly disagree)

1. Troubleshooting transport ventilator issues is relevant to my role as a NICU provider.

1 2 3 4 5

2. This session was helpful in learning how to set up the transport ventilators.

1 2 3 4 5

3. This session was helpful in identifying ventilator problems and learning how to fix them.

1 2 3 4 5

4. Prior to this session, I was confident in troubleshooting a malfunctioning ventilator during transport.

1 2 3 4 5

5. After completing this session, I am confident in troubleshooting a malfunctioning ventilator during transport.

1 2 3 4 5
