# Supplementary material for: Neonatal Transport Ventilation: Simulation to Improve Knowledge and Skills
Source: MedEdPORTAL. 2022 Sep 13;18:11272. doi: 10.15766/mep_2374-8265.11272 (PMC9468152; doi:10.15766/mep_2374-8265.11272)
Supplement: Supplementary file 1 — Simulation Scenarios Guide.docxTransport Ventilator Troubleshooting Visual Aid.pptxPostsession Survey.docxLearner Knowledge Test.docxKnowledge Test Answers.docx [file mep_2374-8265.11272-s001.zip › D. Learner Knowledge Test.docx]

**Appendix D:** Transporter Knowledge Test

Demographics: RT MD NP PA

Pretest 1 Week Posttest 4 Week Posttest 8 Week Posttest

1. You are transferring a 2 kg preterm male who requires NIV/NIPPV via nasal prongs. What flow setting do you choose?

A. Constant flow

B. Flow trigger

2. Your transport team is called to transfer a 0.9 kg preterm female who is intubated on SIMV-PC. What flow setting do you choose?

A. Constant flow

B. Flow trigger

3. You arrive at the referral institution to transport the 0.9 kg preterm infant who is intubated on SIMV-PC. The respiratory therapist has just noticed that the flow sensor is cracked, malfunctioning and unusable. What setting/parameter will you change on the transport ventilator?

_________________________________________________

4. During transport of an intubated infant, the transport ventilator begins to alarm. On the ventilator screen, peak pressure (PIP) is low. What could be causing this (minimum 3 answers)?

_________________________________________________

_________________________________________________

_________________________________________________

5. During transport of an intubated infant, the transport ventilator begins to alarm. On the ventilator screen, peak pressure (PIP) is low. As you begin to troubleshoot the ventilator, the infant begins to desaturate and become cyanotic. You are unable to determine the reason for the alarm. How do you proceed?

_________________________________________________

Answer questions 6, 7 and 8 based on the following:

You have arrived back to your home institution after transporting a full term neonate with meconium aspiration syndrome. The infant is intubated on SIMV-PC with a rate of 40, pressures of 25/5 and 50% FiO_2_. As you unload the infant from the ambulance, you hear a loud, high-pitched noise.

6. What is the source of the noise?

_________________________________________________

7. What do you check next?

_________________________________________________

8. How do you fix the issue (minimum 2 answers)?

_________________________________________________

_________________________________________________

9. How many valves and levers are there in the oxygen and medical air circuit, and where are they located?

_________________________________________________

Answer questions 10 and 11 based on the following:

An intubated preterm infant born at 27 weeks’ gestation weighing 0.9 kg is being transported on SIMV-PC with a rate of 40, pressures of 20/5, pressure support of 5 cmH_2_O, 30% FiO_2_ and a flow trigger of 3 L/min. During the ambulance ride, you note that the ventilator is giving a rate of 60 fully supported breaths per minute. You conclude that the ventilator is auto-cycling.

10. Why is the ventilator auto-cycling?

_________________________________________________

11. What ventilator adjustments can you make to overcome this?

_________________________________________________

12. Your transport team is called to transport a full term, 3 kg infant with meconium aspiration syndrome. Upon arrival to the referring hospital, you begin to set up the transport ventilator. You start by choosing to set Tidal Volume and then select flow trigger mode with a flow sensor in place in the circuit. You set the following parameters: TV 25ml, rate 35, PEEP 6 cmH_2_O, PS 7 cmH_2_O, FiO_2_ 50%. You double-check all of your settings prior to placing the infant on the ventilator and note on the ventilator screen that the inspiratory time is 0.14 seconds, but you are unable to directly increase the iT to 0.35 seconds. What other setting can you adjust to indirectly increase the iT?

_________________________________________________

13. Your transport team is called to transport a full term, 3 kg infant with meconium aspiration syndrome. Upon arrival to the referring hospital, you begin to set up the transport ventilator. You start by choosing to set Inspiratory Time and then select flow trigger mode with a flow sensor in place in the circuit. You set the following parameters: rate 35, PIP 25 cmH_2_O, PEEP 6 cmH_2_O, PS 7 cmH_2_O, iT 0.35 seconds, FiO_2_ 50%. You double-check all of your settings prior to placing the infant on the ventilator and note on the ventilator screen that the delivered TV is 56ml (~19ml/kg), but you are unable to directly decrease the TV. What other setting can you adjust to indirectly decrease TV?

_________________________________________________

Answer questions 14, 15 and 16 based on the following:

You are called to transfer a 1wo preterm infant born at 28 weeks’ gestation with severe respiratory distress syndrome. You determine that the infant should be transported on high frequency ventilation. You set the following parameters: MAP 7, rate 500. When you place the infant on the transport ventilator, you note that he’s jiggling to mid-chest.

14. What is your next step?

_________________________________________________

15. What must you check if you make a change to a high-frequency parameter?

_________________________________________________

16. Fifteen minutes later, the infant begins to desaturate. You look at the high-frequency ventilator screen and note that the measured MAP has dropped to 3. What could be causing this (minimum 3 answers)?

_________________________________________________

_________________________________________________

_________________________________________________

17. Your transport team is called to transfer a full term infant with meconium aspiration syndrome and persistent pulmonary hypertension for ECMO evaluation. You place the infant on the high frequency ventilator with inhaled NO at 20 ppm. Twenty minutes into the transport, you notice that the measured iNO is dropping. What could be causing this (minimum 3 answers)?

_________________________________________________

_________________________________________________

_________________________________________________

18. Your transport team is called to transfer a full term infant with meconium aspiration syndrome and persistent pulmonary hypertension for ECMO evaluation. You place the infant on the high frequency ventilator with inhaled NO at 20 ppm. Forty minutes into the transport, you notice that the infant is no longer jiggling to the level of the groin. You appropriately check the medical air and oxygen tanks and look for leaks in the circuit. There is adequate gas supply, and all circuit connections are tight. You decide to increase amplitude on the high-frequency ventilator with improvement in jiggle to the level of the groin. A few minutes later, you see that the measured NO ppm has decreased. What is your next step?

_________________________________________________

19. During transport of a patient on iNO, you are alerted that the NO_2_ level is 2. What is your next step?

_________________________________________________
