# Supplementary material for: Neonatal Transport Ventilation: Simulation to Improve Knowledge and Skills
Source: MedEdPORTAL. 2022 Sep 13;18:11272. doi: 10.15766/mep_2374-8265.11272 (PMC9468152; doi:10.15766/mep_2374-8265.11272)
Supplement: Supplementary file 1 — Simulation Scenarios Guide.docxTransport Ventilator Troubleshooting Visual Aid.pptxPostsession Survey.docxLearner Knowledge Test.docxKnowledge Test Answers.docx [file mep_2374-8265.11272-s001.zip › E. Knowledge Test Answers.docx]

**Appendix E:** Transporter Knowledge Test Answers

1. A

2. B

3. Change flow trigger to constant flow

4. There is a leak in the circuit, the endotracheal tube is malpositioned, or the medical air and/or oxygen tank(s) is/are empty.

5. Begin positive pressure ventilation with your back-up device (self-inflating bag or T-piece resuscitator)

6. Blender

7. Check medical air and oxygen tanks

8. Next steps depend on which tank is empty. If available, change out the empty tank for a full one. If the medical air tank is empty, you may also increase FiO2 to 100% briefly until you’re able to get the infant to the NICU (must weight risks and benefits of oxygen exposure).

9. Four in total; two each in the medical air and oxygen circuits. Two are located on the tubing leaving the oxygen and medical air tanks, and two are located on the ventilator housing near the blender.

10. The motion of the ambulance (bumps in the road, etc) is triggering breaths in flow trigger mode.

11. Increase the flow trigger above 3L/min until the ventilator rate decreases to 40

12. When setting TV, inspiratory time is dependent on flow rate. As flow is decreased (dial below ventilator screen), iT will increase.

13. When setting iT, TV is dependent on flow rate. As flow is decreased (dial below ventilator screen), TV will decrease.

14. Increase amplitude until you note that the infant is jiggling to the groin. Note that the high-frequency ventilator does not give a numeric measurement of the amplitude.

15. Any time that any high frequency parameter is adjusted, the other parameters must be checked and titrated to maintain desired settings. If amplitude is increased, MAP and rate may also increase and will need to be adjusted down as amplitude is increased to desired level.

16. There is a leak in the circuit, the endotracheal tube is malpositioned, or the medical air and/or oxygen tank(s) is/are empty.

17. The NO tank is empty, the NO valve was inadvertently closed, or there is a leak at the NO supply or sampling line on the Phasitron.

18. Any time that any high frequency parameter is adjusted, the other parameters, including iNO flow, must be checked and titrated to maintain desired settings. If measured NO level drops after adjusting HF ventilator settings, NO flow must be increased to achieve desired ppm.

19. If there is accumulation of NO_2_ in the circuit, the endotracheal tube should be briefly disconnected to bleed out the NO_2_
